# A Young Heart With Coronary Dissection

**DOI:** 10.7759/cureus.23880

**Published:** 2022-04-06

**Authors:** Kelash Kumar, Amit Gulati, FNU Poonam, Shiv Raj

**Affiliations:** 1 Internal Medicine, Wyckoff Heights Medical Center, Brooklyn, USA; 2 Internal Medicine, Maimonides Medical Center, Brooklyn, USA; 3 Cardiology, Maimonides Medical Center, Brooklyn, USA

**Keywords:** coronary stents, cocaine use, cardiac chest pain, spontaneous coronary dissection, coronary artery angiography

## Abstract

Chest pain is one of the common complaints encountered in clinical practice. Multiple diseases present as chest pain and often the etiology can be challenging to diagnose. Among the cardiac causes, coronary artery dissection is one of the life-threatening conditions and is often misdiagnosed as an acute coronary syndrome because of its similar presentation. In this case report, we will share a case of coronary artery dissection, which was initially managed as a non-ST-elevation myocardial infarction. We will share the modalities used to diagnose spontaneous coronary artery dissection and how the management differs between acute coronary syndrome and spontaneous coronary artery dissection.

## Introduction

Coronary artery dissection is an uncommon cause of acute chest pain. It is usually of two types, atherosclerotic and non-atherosclerotic, which is also known as spontaneous coronary artery dissection (SCAD) [1]. Often patient presenting with SCAD lacks standard cardiovascular risk factors [2,3]. Fibromuscular dysplasia, postpartum status, multiparity (≥4 births), connective tissue disorders (Marfan or Ehlers-Danlos syndrome), systemic inflammatory conditions, and hormonal therapy are usually the conditions predisposing to this entity [2,3]. Intense exercise or emotional stress, labor and delivery, intense Valsalva-type activities, recreational drug use, and aggressive hormonal therapy can result in the development of acute SCAD, particularly with underlying arteriopathy [2-4].

## Case presentation

**Figure 1:**
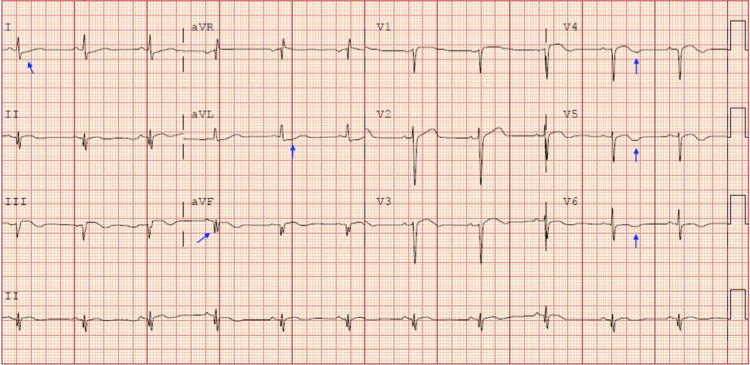
Electrocardiogram showing sinus rhythm with Q waves in L3 and aVF, reciprocal ST depression in L1 and aVL, and T wave inversions in V3-V6. aVF: unipolar augmented vector foot lead; aVL: unipolar augmented vector left lead.

A 39-year-old man with 10 pack-year cigarette smoking history and cocaine use was evaluated in the emergency department for acute onset of constant retrosternal chest pain and tightness associated with diaphoresis and mild shortness of breath. The patient had sniffed two packets of cocaine a day before the onset of symptoms. The patient had never experienced the above symptoms before. There was no history of hypertension, hyperlipidemia, diabetes mellitus, or prior family history of coronary artery disease. On arrival at the emergency department, the patient’s temperature was 38.2°C, pulse rate was 69/min, blood pressure was 144/104 mmHg, respiratory rate was 18/min, and saturation was 99% on room air. Physical examination was notable for young anxious male in mild to moderate distress, otherwise non-significant. EKG was obtained (Figure 1), which noticed sinus rhythm with Q waves in L3 and unipolar augmented vector foot lead (aVF), reciprocal ST depression in L1 and unipolar augmented vector left lead (aVL), and T wave inversions in V3-V6.

Pertinent laboratory studies showed initial troponin of 3 ng/dl, creatinine phosphokinase of 2576 IU/L, and lactic acid of 4.2 mmol/L (Table 1).

**Table 1:** Laboratory results of the patient.

Test	Result	Reference values
Troponin	3 ng/dl	0.00-004 ng/dl
Creatinine phosphokinase	2576 IL/U	24-205 IL/U
Lactic acid	4.2 mmol/L	0.5-1.6 mmol/L

CT angiography ruled out aortic dissection. A bedside echocardiogram showed akinetic mid to apical anteroinferior septum and akinetic mid to apical inferolateral wall with an ejection fraction of 25% (Videos 1, 2).

**Video 1:** Echocardiogram apical view showing akinetic apex and inferior-septal wall with an ejection fraction of 25%.

**Video 2:** Echocardiogram apical 5C view showing anteroseptal wall akinesia.

The patient remained in distress despite morphine, aspirin, benzodiazepines, and intravascular fluids. The patient was started on anticoagulation with IV heparin followed by urgent cardiac catheterization and angiogram, which revealed spiral dissection of the mid-left anterior descending artery (LAD) extending to the apex (Videos 3, 4).

**Video 3:** Cardiac angiogram (RAO caudal view) showing the spiral dissection of the mid-LAD extending to the apex. RAO: right anterior oblique; LAD: left anterior descending artery.

**Video 4:** Cardiac angiogram (anteroposterior cranial view) showing the spiral dissection of the mid-LAD extending to the apex. LAD: left anterior descending artery.

Given the patient was hemodynamically stable and flow was preserved, no intervention was performed and the patient was managed conservatively.

## Discussion

SCAD is known for non-traumatic and non-iatrogenic detachment of components of the coronary arterial wall, which results from hematoma formation inside tunica media and spreads circumferentially and longitudinally to create a false lumen, which causes compression of the true lumen and leads to coronary insufficiency, myocardial infarction, and ventricular arrhythmia [1-5]. SCAD comprises almost 4% of total patients presenting with acute coronary syndrome (ACS), out of which women aged <50 years account for 35% of cases [2,3]. SCAD is a rare cause of ACS in men and the old age population [4].

Clinical diagnosis of SCAD could be challenging, as presenting symptoms are no different from other causes of ACS, and no chemical biomarker is available that distinguishes SCAD from atherosclerosis [1]. Fibromuscular dysplasia or connective tissue diseases, pregnancy-associated ACS, cocaine use, and non-responsiveness to intracoronary nitrates favor SCAD over atherosclerosis [5].

Cocaine is known for its sympathomimetic properties and leads to many non-atherosclerotic coronary effects, which include an increase in heart rate, blood pressure, oxygen demands, wall stress, and dissection [6]. Cocaine is a rare but well-recognized cause of SCAD, especially among young adults [7].

Both invasive and non-invasive management of ACS caused by SCAD or atherosclerosis varies, which highlights the criticalness of accurate diagnosis [1-6]. Coronary angiography is a commonly used modality for the diagnosis of SCAD and the classical intimal flap represents the hallmark of this disease [6].

The friable and disrupted nature of the coronary vessel wall in SCAD could produce the worst outcomes for percutaneous coronary intervention as compared to atherosclerotic etiology of coronary disease. Both European and American consensus documents recommend against re-vascularization in hemodynamically stable patients with maintained distal flow in the culprit coronary artery without demonstrable ongoing ischemia [8].

SCAD and atherosclerotic coronary artery disease differ in the pathophysiology, mechanism of ischemia, and outcomes of percutaneous coronary intervention, hence using the standard ACS treatment in SCAD could impose potential risk. For instance, the use of anticoagulation in ACS offers protection by reducing clot burden but hypothetically could aggravate the bleeding and propagate the dissection in SCAD. Hence, if systemic anticoagulation is initiated on a presentation for suspected ACS, consideration of discontinuation is appropriate once SCAD is diagnosed and in the absence of other indications for systemic anticoagulation [9].

The use of guideline-based dual antiplatelet therapy is indicated for patients with SCAD who underwent revascularization with stenting. However, data on dual antiplatelet in patients not undergoing coronary intervention are lacking and require further clinical trials [8]. Statins are not indicated for the treatment of SCAD [2]. Standard heart failure medications are indicated for left ventricular dysfunction, and hypertension should be treated [9].

## Conclusions

SCAD can lead to coronary insufficiency, myocardial infarction, ventricular arrhythmia, and sudden death. Among many etiologies, cocaine is a rare but well-recognized cause of SCAD, particularly among young adults. Cocaine was the obvious cause of SCAD in our patient.

Immediate coronary angiography should be considered to exclude the diagnosis of SCAD in patients presenting with clinical features indistinguishable from acute coronary syndrome but at low risk of atherosclerotic acute myocardial infarction, in particular young to middle-aged women. The optimal management remains to be defined. In hemodynamically stable patients with maintained coronary flow, a conservative management strategy is preferred because the percutaneous coronary intervention is associated with high complication rates, so as the emergency coronary artery bypass surgery, even in those patients presenting with preserved vessel flow. Revascularization does not decrease the risk of recurrent SCAD.
